# Exploring udder functional morphology in nucleus herd Cyprus Damascus goats and its effects on milk yield

**DOI:** 10.1016/j.vas.2026.100743

**Published:** 2026-06-17

**Authors:** P. Savvides, G. Hadjipavlou

**Affiliations:** Animal Production Section, Agricultural Research Institute, 1516 Lefkosia, Cyprus

**Keywords:** Udder morphology, Functional morphology, Damascus goats, Breeding selection, Milk yield

## Abstract

The growing interest in increasing milk yield in dairy goats and concurrently environmental concerns and societal demands, favor more sustainable farming. In this study, we aimed to detect the effects of quantitative and qualitative udder morphological parameters on milk yield in order to develop a weighted index that could predict milk yield in Cyprus Damascus goats from a nucleus herd. We recorded morphological parameters from goats during lactation (N = 84) and during their dry period (N = 72). For milk yield, data for three consecutive years (2022–2024) were used. Teat positioning in dry individuals, was the only qualitative parameter deemed significant for milk yield, with teats positioned horizontally having positive effects on milk yield. Morphological parameter evaluation showed that the dry group maintained more stable udder morphology and further analyses were focused on this group. Udder morphometry was significantly different across different age groups; therefore, age groups were analyzed separately to define their morphometric index. Moreover, we detected different effects for each age group; for younger individuals, teat-related parameters appeared significant, while for older individuals, parameters of the lower udder were deemed significant. The developed weighted indices for each age group provided accurate predictions, given the complexity and multivariability of biological functional systems. The proposed indices can be used as the basis for further exploration of traceable morphological correlations in younger goats, with bigger samples and incorporation of genetic data. Such an approach may provide a highly effective method to assist sustainable dairy goat breeding programs.

## 1 Introduction

Small ruminant milk yield, has been a decisive factor in breed selection, especially in modern days, due to prestigious and highly marketable cheese types (e.g., halloumi, feta, pecorino romano) (Mandolesi et al., 2024). Insufficient amounts of milk and hence dairy products, could cause loss of income and a significant reduction in the stakeholders’ well-being and quality of life (Chniter et al., 2024; Herrero et al., 2009; Vargas Bello Pérez et al., 2022).

Despite the financial and cultural importance of goat farming, there are also potential negative effects due to greenhouse emissions per milk unit, especially in view of the increasing goat population worldwide (Danieli & Ronchi, 2018; Olmo et al., 2024). At the same time, ethical concerns related to ruminant feed needs, and well-being, have been raised (Pulina & Bertoni, 2023; Pulina et al., 2017; Ripple et al., 2014). The most efficient and direct way to tackle the aforementioned issues is the increase of per-animal-derived milk yield through breeding programs (Laborde et al., 2021).

In this study, we focused on Cyprus Damascus goats (ARI nucleus herd), a breed that was developed via systematic genetic evaluations, after being imported 90 years ago to Cyprus to improve the milk yield of indigenous breeds (Mavrogenis et al., 2006). The breed makes up the 23% of the whole island’s goat population. Despite its high impact and high milk yield, double in volume to that of Cyprus Chios sheep (El Hag et al., 1995), there are limited studies systematically documenting its milk yield improvement. Moreover, the fact that Cyprus is obligated to increase its sheep and goat milk production to fulfil the registration criteria for halloumi cheese, a Cyprus PDO product (Dimitriou et al., 2024), highlights the necessity for the breed’s milk yield improvement.

While breeding programs for the main small ruminant commercial breeds mostly rely on genetic and/or genomic evaluation of milk traits (Barillet, 2007; Massender et al., 2022; Negro et al., 2024), there are also recent studies where attention has been redirected towards solely phenotypic evaluation (Billah et al., 2025) and more specifically focused on the improvement of udder morphology (Marshall et al., 2024; Pierre-Guy et al., 2024). It has been well established that udder morphology affects milk yield due to its association with milk-related genetic components or due to mechanical variables facilitating the milking procedure (Margo et al., 2024; Marshall et al., 2024). However, despite the importance of Damascus goats, the only available research on the udder morphological effects on milk yield, from Cyprus or elsewhere, is that of Mavrogenis et al. (1989), which was carried out 36 years ago.

The association of various quantitative and qualitative udder traits with milk yield in goats and sheep has been extensively explored (Emediato et al., 2008; Erduran et al., 2021; Mavrogenis et al., 1989). Even so, the number and combination of recorded parameters, the measurement procedures, and their association with milk-related traits and consequent interpretation of results largely vary across species and breeds.

Herein, we aimed to develop a novel and accurate udder morphological index for the Cyprus Damascus goats of ARI nucleus herd, which could potentially allow the selection of high milk-yield individuals, considering also the udder ontogenetic allometry. Regarding the latter, although in many studies, udder measurements have been recorded during the lactation period (Capote et al., 2006; Vrdoljak et al., 2020), machine milking and suckling may lead to variable mechanically-induced alterations across lactations. Such alterations would apply predictive restrictions on any given index’s repeatability. To handle the ontogenetic changes, we attempted to evaluate the optimal period for udder measurements. Moreover, we propose the utilization of the multimodel inference approach as the most appropriate evaluation method; a robust and commonly used method in zoological and functional morphology studies for the examination of performance and relevant morphological variables (Burnham & Anderson, 2004; Crittenden et al., 2023; Savvides et al., 2019).

The objectives of this study were the following:

To examine udder morphological parameters from different udder states (i.e., lactation vs dry period) and evaluate their suitability as milk yield predictors based on the repeatability of their measurements. The udder state with the least morphological variability, free from exogenous interferences, would be considered the most appropriate for stable parameter recordings and further analyses.

1. To search for any morphological or milk yield differences among age groups.
2. To test which of the recorded quantitative and qualitative morphological traits are significantly associated with milk yield across lactations, and among age groups, utilizing the multimodel inference approach.
3. To pursue the development of an age-specific weighted index able to predict the Cyprus Damascus goat milk yield.

## 2 Materials and methods

This study was carried out using measurements on Cyprus Damascus goats at the Agricultural Research Institute’s (ARI) Experimental Farm (Athalassa area, Nicosia, Cyprus). The ARI nucleus herd consists of approximately 300 individuals (milked twice per day), which, since 2008, have been used to supply commercial farms with improved individuals. Full productivity, management, pedigree, and health information were routinely recorded for these animals, as they are part of the ARI breeding nucleus.

### 2.1 Study design and grouping

**Fig. 1:**
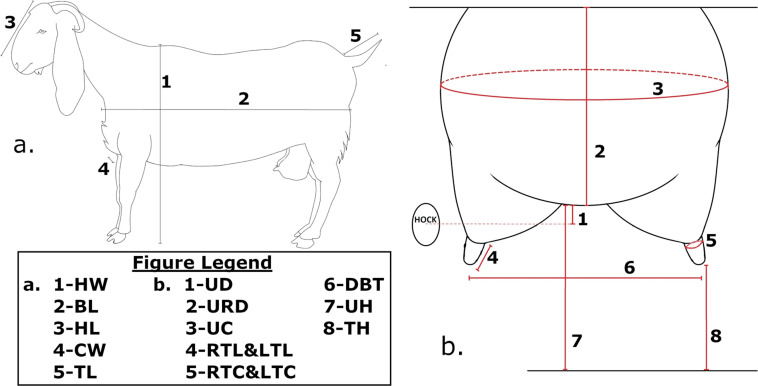
The morphological parameters were recorded for each individual. Description of each parameter from Fig. 1a and abbreviations: 1. height at the withers, HW (ICAR, 2025), 2. body length, BL (Esquivelzeta et al., 2011), 3. head length, HL (Esquivelzeta et al., 2011), 4. chest width, CW (ICAR, 2025) and 5. tail length, TL (Khandoker et al., 2016). Description of each parameter from Fig. 1b and abbreviations: 1. udder depth, UD (ICAR, 2025), 2. udder rear depth, URD (Emediato et al., 2008), 3. udder circumference, UC (Emediato et al., 2008), 4. right and left teat length, RTL and LTL (Mavrogenis et al., 1989), 5. right and left teat circumference, RTC and LTC (Wang, 1989), 6. distance between teats, DBT (Wang, 1989), 7. udder height, UH (Wang, 1989), 8. teat height, TH, and 9. udder volume (UV) (Emediato et al., 2008).

In this study, we attempted to develop an all-rounded approach through the incorporation of various morphological parameters selected from different studies, as shown in Fig. 1a and b. The datasets we used regarding the morphological parameters were obtained in 2024, from Cyprus Damascus goats divided into two different groups. The first, or two-staged group, refers to individuals (N = 42) whose morphological parameters were recorded twice during their lactation period: postpartum (∼15 days after parturition) and post-weaning (∼45 days after parturition) (84 measurements in total). This group was used to evaluate any possible changes in udder morphology during the lactation period and to determine if the lactation period can provide consistent morphological data. The second, or dry group, refers to different individuals (N = 75), where the morphological measurements were recorded during their dry period. Whenever the analysis restrictions allowed it (i.e., annual milk yield comparison, litter size, and rearing method), we pooled the data, and statistical analyses were performed in one mixed group (N = 117).

Milk yield for each individual (milk yield total and milk yield at 60 days) was calculated for three consecutive years (2022, 2023, and 2024), following the method of VanRaden (1997). Also, we retrieved data for each individual regarding the age (which is directly related to parity), the rearing method, and the litter size, and whenever necessary, they were categorized accordingly. Categories were as follows: For age: Group A = 1–2 years (N = 19), Group B 3+ years (N = 56); For rearing method: suckling, no suckling, unknown; For litter size: total number of kids at birth.

### 2.2 Morphological recordings

First, due to the multiple pressures the udders experience during the lactation period (suckling, machine milking), we wanted to examine the udder morphology changes throughout that period. Following the methodologies of Mavrogenis et al. (1989), Wang (1989), and Emediato et al. (2008), we recorded the morphological measurements, as shown in Fig. 1a and b. For the purpose of examining the extent of udder changes, we recorded the parameters in two periods: a) 15 days postpartum and b) 45 days postpartum (two-staged group, N = 42). Using the Wilcoxon signed-rank test, we examined the significance of udder transformation pre- and post-weaning within one lactation period and of differences in milk yields across different lactations.

**Fig. 2:**
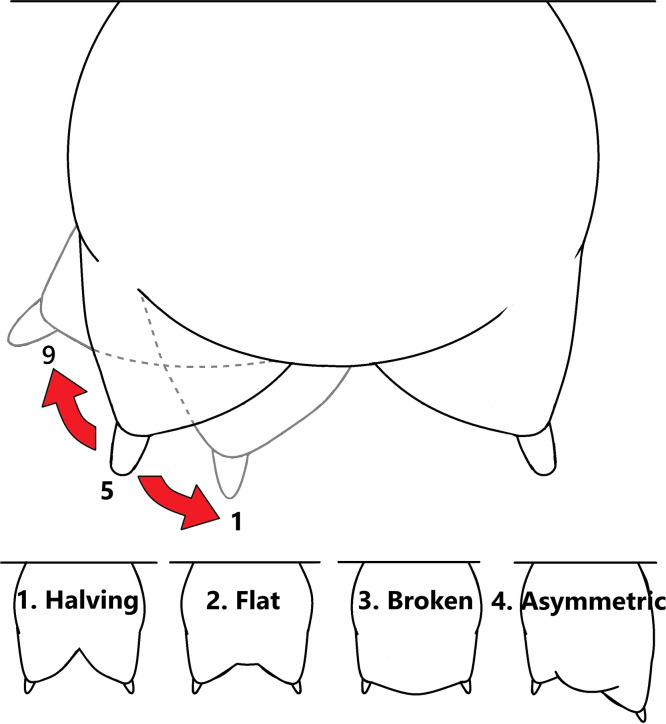
Qualitative parameters were recorded for each individual based on udder conformation and teat positioning, as shown in the figure.

Second, following the methodologies of Mavrogenis et al. (1989) and guidelines provided by ICAR (2025) regarding udder conformation and teat positioning (Fig. 2), we recorded some qualitative udder traits (udder conformation, right and left teat positioning) and parturition traits (litter size and rearing method: suckling vs no suckling) to assess their effects on milk yield.

### 2.3 Statistical analysis

Before any further analyses, we used the formula of Lleonart et al. (2000), as shown below, in order to remove any size-related effects from the morphological variables. Outliers were also removed.

$$Ya=Yi*{\left(Xm/Xi\right)}^{b}$$

where Yi: the observed value of each morphological parameter from each individual, Xm: the average body length of each group, Xi: the body length of each individual, and b: the slope of the regression of each morphological parameter against body length.

Using the Wilcoxon signed-rank test, we compared each individual’s morphological parameters (two-staged group), and milk yield across lactation periods (2022, 2023, and 2024) (both groups).

In order to evaluate the association of milk yield (milk yield total and milk yield 60) for three consecutive years (2022, 2023, and 2024) with litter size, rearing method, udder conformation, and teat positioning, we used general linear models. For litter size and rearing method analyses, we used the mixed group (N = 117). For the udder conformation and teat positioning analyses, because of the different udder states (postpartum, post-weaning, and dry/relaxed), we analyzed the two-stage and dry groups separately. Using the same grouping approach, we used Kruskal-Wallis tests to examine any significant differences in milk yield among the different categories of litter size, rearing method, udder conformation, and teat positioning for each lactation period (2022, 2023, and 2024).

Next, we used PERMANOVA (post hoc Dunn test) to examine whether there are any milk yield differences between age groups. Moreover, we performed PERMANOVA to search for any age-related differences across the morphological parameters (mixed). We tested for multicollinearity among the morphological parameters, and then we utilized the multimodel inference approach (MUMIN package on R, Barton, 2022) in order to examine the effect of each morphological parameter on goat milk yield. To evaluate the relative importance of each variable within the multimodel inference framework, we used the sum of Akaike weights (Giam & Olden, 2016).

Given the results of the multimodel inference for the udder morphological parameters, we designed a milk yield predictive index based on the weighted effects of the significant parameters (coefficients and relative importances) for each group. The strength of the index was tested and evaluated based on its performance in linear models against the milk yield and its Akaike Information Criterion (AIC) scores.

## 3 Results

### 3.1 Morphological and milk yield examination for the two-stage and dry groups

As shown in Table 1, the results of the Wilcoxon signed-rank test showed that 6 out of 11 udder parameters from the two-staged group differ significantly, with the post-weaning measurements showing higher values, indicating alterations, probably due to the suckling and/or machine milking effects. Wilcoxon signed-rank test showed no significant differences in milk yields across lactations (2022, 2023, and 2024) for both the two-stage and the dry groups.

**Table 1:** The Wilcoxon signed-rank test results only for udder morphological parameters from the two-stage group (n = 42), that showed significant differences. PP: post-partum, PW: post-weaning.

Parameter	N	Mean (cm)		SD (cm)		p-value
		PP	PW	PP	PW	
RTL	133	3.21	3.85	0.89	0.90	<0.001
LTL	189	3.30	3.82	0.85	0.90	<0.005
LTC	280	5.50	5.88	0.97	1.04	<0.05
DBT	255	18.59	20.47	3.98	4.54	<0.05
UH	169	29.1	31.84	4.58	4.01	<0.001
TH	173	24.40	26.95	6.30	5.74	<0.001

RTL and LTL: right and left teat length, LTC: left teat circumference, DBT, distance between teats, UH: udder height, TH: teat height.

### 3.2 Qualitative data results

No significant associations (linear regressions) or differences (Kruskal-Wallis test) were detected when examining milk yield against litter size, and rearing method for the mixed group across lactation periods. In addition, no significant association was found between milk yield and udder conformation for both the two-stage and dry groups. However, the positioning of both the right (RT) and left teat (LT) from the dry group appeared to have significant effects on milk yield, only for year 2024 (Table 2). Similarly, the data for the same individuals from year 2024, yielded significant differences when they were categorized based on their teat positioning (Table 2). More specifically, the teats positioned horizontally with respect to the ground and higher on the udder body were associated with higher milk yield.

**Table 2:** Linear regression and Kruskal-Wallis results to examine associations and differences for the dry group’s milk yield in 2024 and teat positioning (n = 75). Mean milk values are given in kg.

Linear model			F _(1,73)_	R^2^	Coefficient		p-value	
Milk yield total∼		RT	6.436	0.081	19.86		<0.05	
Milk yield 60∼		RT	10.1	0.121	10.538		<0.005	
Milk yield total∼		LT	6.685	0.083	12.332		<0.05	
Milk yield 60∼		LT	10.57	0.126	6.558		<0.005	
Kruskal-Wallis tests								
Parameter	Positioning		Mean milk total (SD)	Mean milk 60 (SD)	χ^2^		p-value	
					Milk total	Milk 60	Milk total	Milk 60
RT	1 (n = 20)		153.02 (61.8)	71.2 (28.48)	7.27	10.23	<0.01	<0.005
	5 + 9* (n = 55)		199.89 (79.8)	98.6 (32.8)				
LT	1 (n = 22)		151.9 (63.2)	72.4 (27.9)	8.2	9.73	<0.005	<0.005
	5 + 9* (n = 53)		201.24 (79.6)	98.6 (33.26)				

RT: right teat, LT: left teat. Data for positions 5 and 9 were pooled due to the lack of significant differences between them.

### 3.3 Multimodel inference results

**Fig. 3:**
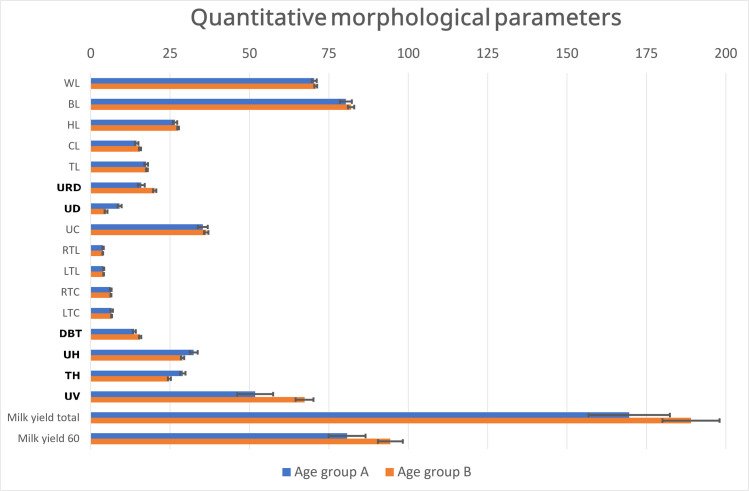
The averaged values of each quantitative morphological parameter for both age groups. Descriptions and abbreviations of each parameter, are given in Fig. 1. All morphological parameters are in cm except UC (cm^3^) and Milk yield total and 60 (kg). UV values are divided by 100 for visualization reasons. The parameters with significant differences between age groups are presented in bold.

For the evaluation of the morphological effects, we chose to analyze data only for the dry group, since the udder exhibited significant alterations during the lactation period (two-staged group), preventing the detection of a stable predictive pattern. In order to prepare the data and specify the data grouping approach to be analyzed, we searched for differences between age groups. PERMANOVA test showed no statistically significant differences in milk yields between the two age groups. However, the morphological parameters differed significantly between the two age groups (Fig. 3. and Table 3). Based on the results shown in Table 3, we performed the multimodel inference analyses for milk yield total and milk yield 60 from 2024, separately for Age group A (N = 19; age 1–2 years) and Age group B (N = 56; age 3 years and above). The results of the multimodel inference, for both age groups, are shown in Table 4.

**Table 3:** PERMANOVA and Dunn test results on the differences in morphological parameters based on age groups. Only significant differences are shown. Abbreviations for the morphological parameters are given in Fig. 1. Mean values are in cm except UV which is in cm^3^.

Parameter	Age group A (1–2 years old, n = 19)		Age group B (3+ years old, n = 56)		F	p-value
	Mean	SD	Mean	SD	9.513	<0.005
					Dunn test	
					Z	p-value
URD	15.93	4.47	20.14	4.10	−3.3178	<0.001
UD	9.10	3.02	4.83	3.35	4.3612	<0.001
DBT	13.70	1.86	15.55	3.03	−2.5521	<0.05
UH	32.43	4.90	28.92	3.83	2.7288	<0.01
TH	28.90	3.49	24.76	3.79	3.5267	<0.001
UV	5175.06	2718.75	6732.91	2192.46	−2.8870	<0.01

URD: udder rear depth, UD: udder depth, DBT: distance between teats, UH: udder height, TH: teat height, UV: udder volume.

**Table 4:** The results of the multimodel inference analyses. Non-significant parameters or parameters appearing in models with a ΔAIC <2 are not presented in the table. F and R^2^ values refer to the models containing all the parameters from the global model (full/adjusted/conditional). Also, only the values of unstandardized (b) and standardized (β) coefficients for significant parameters are shown in the Table. RI: relative importance. Abbreviations for the morphological parameters are given in Fig. 1.

Milk yield total			
Age group A (n = 19) F = 3.441, R^2^=0.8731/0.6194/0.6964			
Parameter	b (RI)	β (RI)	p-value
RT	34.204 (1)	0.7183 (1)	<0.01
Age group B (n = 56) F = 2.689, R^2^=0.4542/0.2853/0.3886			
Parameter	b (RI)	β (RI)	p-value
UD	−11.707 (1)	−0.4620 (1)	<0.005
UH	6.553 (0.87)	0.296 (0.87)	<0.05
RTL	35.42 (0.06)	0.381 (0.06)	<0.05
Milk yield 60			
Age group A (n = 19) F = 2.261, R^2^=0.6011/0.8189/0.4567			
Parameter	b (RI)	Β (RI)	p-value
LTC	−5.557 (0.5)	−0.399 (0.5)	<0.05
RTL	10.49 (0.72)	0.396 (0.72)	<0.05
TH	−3.96 (0.64)	−0.609 (1)	<0.01
Age group B (n = 56) F = 2.094, R^2^=0.3933/0.2055/0.3311			
Parameter	b (RI)	β (RI)	p-value
UD	−4.2626 (1)	−0.3929 (1)	<0.01
UH	3.015 (0.95)	0.318 (0.95)	<0.05
URD	3.577 (1)	0.4008 (1)	<0.01

RT: right teat, UD: udder depth, UH: udder height, RTL: right teat length, LTC: left teat circumference, TH: teat height, URD: udder rear depth.

**Fig. 4:**
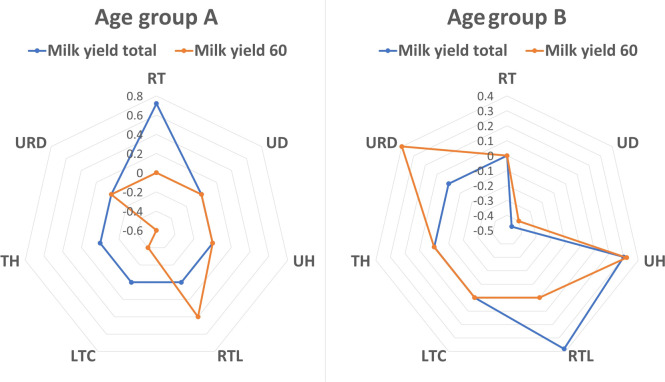
The radar charts show the effect of each significant morphological parameter on the milk yield performance, according to their standardized (β) coefficients from the multimodel inference analyses. The results for age groups A and B are given separately.

Using multimodel inference, we performed two separate analyses for each age group, representing two different sets of morphological parameters. The first set included all the parameters we recorded, and the second set included only the udder-related parameters. In both sets, we excluded udder volume (UV) to avoid multicollinearity issues. The significant predictors for both sets were the same, sharing similar effects. Herein, we present only the results for the udder-related parameters, since they apply towards a more parsimonious concept, they are simpler to record (fewer parameters), and they provide marginally better indices. As shown in Fig. 4., and Table 4, the physiological mechanism affecting milk yield in Cyprus Damascus goats is multivariate and age-dependent. The analysis for age group A revealed right teat positioning (RT) as the only significant morphological parameter for milk yield total. For milk yield 60 of the same group, right teat length (RTL), left teat circumference (LTC), and teat height (TH) showed significant effects. In the case of age group B, udder depth (UD), udder height (UH), and right teat length (RTL) had significant effects on milk yield total, while udder depth (UD), udder height (UH), and udder rear depth (URD) showed significant effects on milk yield 60 (Fig. 4 and Table 4).

### 3.4 Index development and performance

Our main aim was the development of an index with increased robustness for milk yield predictive capacity. Using our findings from the multimodel inference results, we developed two weighted morphological indices (one for each age group) based on the mean values of the standardized, conditional coefficients (β) and relative importances (RI) of the significant parameters for both milk yield total and yield 60. In the formulas below, we present the method we followed for index estimation for age groups A and B.

Age group A:

A=TR*0.36+RTL*0.143+LTC*−0.100+TH*0.305

Age group B:

B=UD*−0.4275+UH*0.2797+RTL*0.0114+URD*0.2004

Next, we regressed the averaged values of milk yield total and 60 with the formulas’ outcome (A and B) and UV values. Then we used the coefficients to develop a final weighted index incorporating all the significant morphological parameters and the UV estimations, following the formulas below.

IndexforagegroupA:A*17.22+UV*−0.004

IndexforagegroupB:B*16.51+UV*0.006

**Fig. 5:**
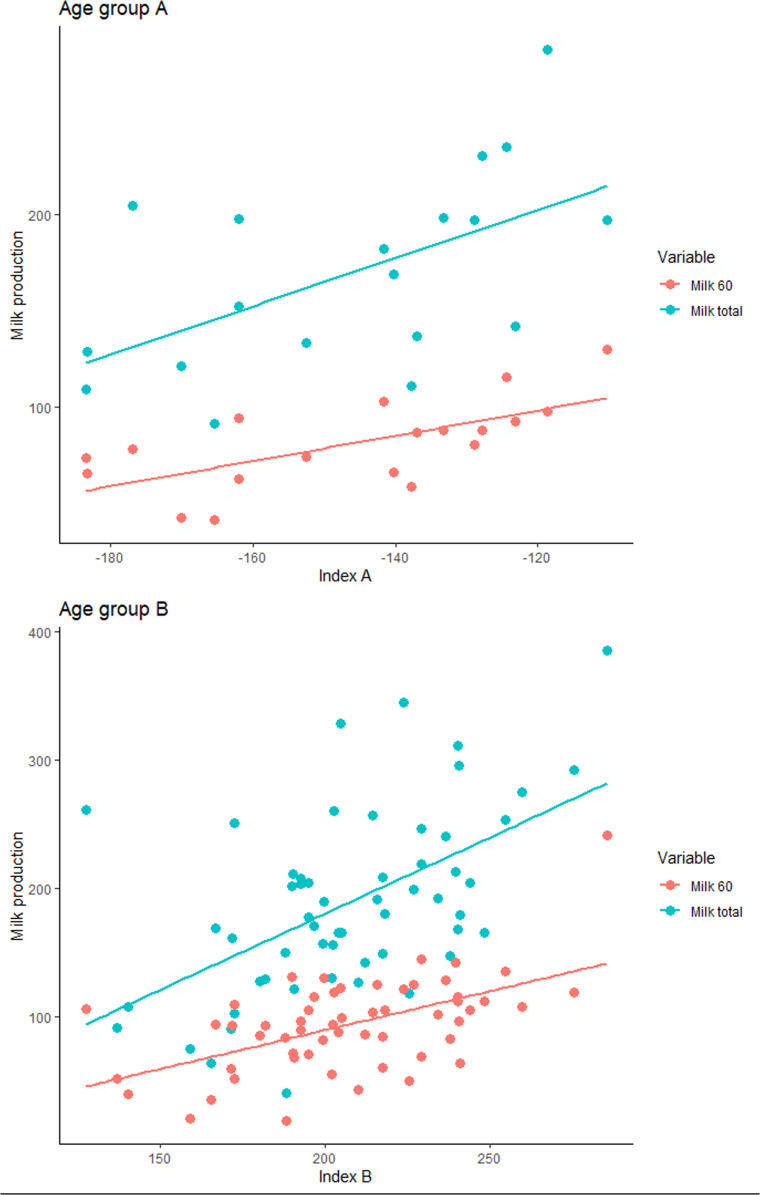
Scatter plots showing the relationship between milk yield total and milk yield 60, and the produced indices for each age group.

We used linear regression to evaluate the predictive capacity of each index against milk yield total and milk yield 60 for each age group (Fig. 5). Both indices performed well for both yield milk total and milk yield 60 (Table 5.). For comparative reasons, we also estimated the AIC values for the best models as shown in Table 5, utilizing the parameters indicated from the multimodel inference results.

**Table 5:** Regression analysis results of indices against milk yield total and milk yield 60. AIC is the index score and BM AIC the score of the best model according to the multimodel inference results.

Age group A (n = 19)		F	R^2^	Coefficient	p-value	AIC	BM AIC
Milk yield total∼	Index A	7.694	0.2994	1.254	<0.05	202.79	214.75
Milk yield 60∼		12.84	0.4164	0.6570	<0.005	168.49	176.85
Age group B (n = 56)		F	R^2^	Coefficient	p-value	AIC	BM AIC
Milk yield total∼	Index B	24.93	0.3119	1.4469	<0.001	640.57	648.59
Milk yield 60∼		23.31	0.2976	0.6052	<0.001	546.71	554.03

## 4 Discussion

### 4.1 Optimal udder morphological recording period

In this study, we found that udder morphological parameters in Cyprus Damascus goats from ARI nucleus herd, show significant changes during the lactation period (postpartum and postweaning comparison). These changes indicate an underlying mechanism, stretching the teat-related parameters and widening the teat-adjacent udder body. This alteration increases the distance between the teats and udder-floor ground clearance. Our results are in accordance with the findings presented in El Sayed et al. (2009), where it was found that significant udder changes take place during lactation in Damascus goats. Moreover, similar findings were recorded in other goat breeds, where an early size increase in directly and indirectly associated secretory tissues (alveoli, parenchymal, stroma), was documented, followed by their decrease in late lactation stages (Ayadi et al., 2011; Lerias et al., 2014; Peris et al., 1999). Despite our observations regarding udder alterations, it is not possible to detect the etiology of these changes, since they can be the outcome of udder physiological mechanisms or mechanical exogenous effects, or both, driven by multiple unstandardized variables. Due to this instability and inability to predict the level of change, we consider the lactation period as a non-optimal udder stage to record and assess morphological parameters. Therefore, despite the absence of repeatable examinations of the udder morphology for the dry individuals in this study, we considered the dry period less likely to be affected by exogenous factors leading to morphological inconsistencies. For this reason, we suggest the dry period as the most appropriate udder state to be evaluated in similar studies. The utilization of only dry individuals, together with the size correction approach we applied to the morphological parameters, increases the accuracy and repeatability of our method and strengthens the outcomes of this study.

Moreover, the teat positioning-milk yield relationship, documented only for the dry group, further enhances the argument regarding the choice of dry-related parameters as the most appropriate predictors for milk yield models. The high heritability potential of teat-related parameters observed in Cyprus Damascus goats (Mavrogenis et al., 1989) renders such parameters efficient candidates to be used in breeding programs.

### 4.2 Effects of qualitative traits on milk production

In the past, it was found that the udder floor conformation of Cyprus Damascus goats from ARI, was associated with milk yield (Mavrogenis et al., 1989). However, in our study, we did not detect such a relationship. Similarly, the effects of other traits such as litter size and rearing method, on milk yields, from three consecutive lactations, were also insignificant. Nevertheless, there was a striking relationship between milk yields and teat positioning within the latest recorded lactation period (2024) for the dry group. This indicates that higher-placed teats (horizontally oriented) are associated with higher milk yields. Even though, to the best of our knowledge, there are no known equivalent studies regarding the role of teat positioning for Damascus goats, it has been documented in the past that teat-associated parameters may affect milk yield in other goat and sheep breeds. More specifically, in Fernandez et al. (1997) (Churra sheep), Manfredi et al. (2001) (Saanen and Alpine goats), Legarra and Ugarte (2005) (Latxa sheep), and McLaren et al. (2016) (Saanen goats), vertically oriented teat placement was associated with higher milk yield. However, Capote et al. (2006) found contradictory results in Tinerfeña goats, where there was a positive relationship between milk yield and residual milk, and the distance between teats. In the absence of any significant effects related to teat length, the increase in the distance can be attributed to the higher positioning of the teat on the udder’s body. This results in an increased spacing between teats and thus difficulties in unhindered milk flow during milking procedures due to the teats’ horizontal position. Similar results have also been documented in sheep breeds (Dzidic et al., 2019). In the literature, it has been documented in other breeds, that horizontally placed teats or teats positioned higher from the ground, resulted in lower somatic cells and risk for mastitis (Adhikari & Malla, 2025; Gabr et al., 2021). Nevertheless, it has to be mentioned that there are considerations regarding the machine milking efficiency due to the horizontal placement of the teats and the consequent inability to secure latching of the milking unit and the extraction of milk below the teat height (Capote et al., 2006; Dzidic et al., 2019). The inability during milking to empty the udder, causes milk stasis, which increases the risk of infections (Peris et al., 1999). In the case of the Cyprus Damascus goats, even though no data on residual milk were available in this study, we detected clear benefits in terms of milk yield if the teats were placed higher on the udder body. Consequently, this finding allows the hypothesis for further increasing the milk yield and lowering the risk of mastitis, through simple mechanical adjustments (i.e., Sagi hook, Dzidic et al., 2019), offering a better anatomical shape for machine milking.

### 4.3 Udder morphology and milk performance

During the examination of udder morphology, we found that older individuals had significantly larger udders without this size difference being correlated with higher milk yield. The detection of age-specific differences and the separate treatment for further evaluation is a significant aspect of our study that allowed the proper grouping of the data. This age-related variation is in accordance with ICAR recommendations regarding the recording of traits in the first lactation period, to secure a higher quality of data (ICAR, 2025). Moreover, considering the younger individuals in the yearling stage, this separation allowed the detection of variance possibly related to the underdeveloped udder (Prabowo & Garip, 2024). Indeed, it seems that this age-related morphological variation results in distinct functional mechanisms related to milk yield in the two age groups. More specifically, teat-related parameters were found to be the best predictors for age group A, while in the case of age group B, parameters mostly related to the lower udder body appeared to be significant. Nevertheless, in both cases, the suggested models provided sufficient support for their predictive power regarding milk yield, highlighting the importance of udder functional morphology in selection programs. Despite the aforementioned important results, it should be noted that there are also other important variables co-regulating milk yield in Cyprus Damascus goats (e.g. environmental and genetic factors) that were not included in our models.

It has been well documented that the effects of udder-related parameters on milk yield can vary across different breeds and species (Dzidic et al., 2019; Kukovics et al., 2006; Vrdoljak et al., 2020) and in Cyprus Damascus goats, such studies were missing. To the best of our knowledge, this is the first study utilizing the multimodel inference approach to explore the relationship between morphology and milk yield not only in goats but in ruminants overall. Such an approach provides significant benefits against other methods used in the past and enhances the predictive accuracy and trust in the resulting models (Burnham & Anderson, 2004; Giam & Olden, 2016; Symonds & Moussalli, 2011). Multimodel inference is effective in complex functional mechanisms such as those affecting milk yield, due to the examination of all possible combinations of models, instead of a single “best” model found in alternative methods, and utilizes AICs for model selection. The produced averaged coefficients among various good models, the degree of representation of each variable separately (RI), and the reduced possibility of model overfitting enable accurate evaluation of the effects of each independent variable.

In the future, we plan to increase our sample, to include individuals from other herds of Cyprus, and associate our findings with genetic data, thus enhancing the accuracy and effectiveness of our method. Furthermore, data on udder morphology during the dry period can be utilized to investigate possible relationships with morphological parameters of pre-lactation young individuals, to explore the possibility of detecting milk-wise high-quality individuals at earlier ages. Such associations may be explored in relation to body height parameters that have been documented for selection programs in other ruminant species (Prabowo & Garip, 2024).

### 4.4 Udder morphological index - a tool for current and future breeding selection

The development of a novel, weighted, and controlled index (averaged conditional coefficients and RI), based on our results, proved to be an accurate and effective milk predictive tool, at least for the Cyprus Damascus goats from the nucleus herd of ARI. More specifically, our indices for both groups appeared to be, overall, better predictors of both milk yield total and milk yield 60, when compared with the “best” models incorporating only the significant parameters. The index models scored higher F-values, standardized coefficients, and lower AIC scores, and occasionally better R^2^. The observed seemingly lower explained variance for the index models (R^2^), compared to those from the global models, can be attributed to overfitting due to the presence of a large number of independent variables, leading to unrealistically good global models. Moreover, R^2^ values ∼0.30 in complex biological systems are often considered to be more reasonable and accurate, due to the multiple parameters influencing such mechanisms and the technical difficulties to monitor all of them (Gygi et al., 2023; Mazor et al., 2016; Moller & Jennions, 2002).

The proposed simple and effective method for index estimation and utilization provides fine ground for further development, which could be easily applied to other breeds and species. This preliminary work is thus not only relevant for Cyprus, but it can serve as a paradigm for the evaluation of other goat or sheep breeds and contribute to the development of sustainable farm systems in Cyprus or other regions with similar climatic conditions (Almasri, 2022; Mahmoud & El Zubeir, 2024). Moreover, we have to mention the importance of bucks in breeding programs. Despite the lack of documented influence in Damascus goats, it is well established that in other breeds, bucks may influence the udder conformation of their female offspring (Jiang et al., 2022; McLaren et al., 2016; Yao et al., 2025), rendering their role crucial for the udder selection and future studies on udder morphology and function.

### 4.5 Limitations

The recorded traits were taken by individuals from the ARI nucleus herd, which has been under selection programs to increase milk and growth rate productivity. Since 2008, it is considered as a genetically improved nucleus providing animals to commercial farms. However, since our recordings are restricted on the nucleus herd and in the absence of external recordings and index validation, it is possible that the observed parameters, udder function, and index performance vary from those of commercial farms. Moreover, the absence of residual milk recordings could affect the milk yield 60 and total milk yield total of each individual, altering the current understanding of udder function. Also, the relatively small sample size of age group A could affect the level of significance of the variables and the index predictive accuracy.

Despite our attempts to collect and use as much data as possible, we must acknowledge that developing a perfect prediction model is impossible. There is always the possibility that missing confounding factors, such as the direct parity association with morphology, the body condition score, and genetic and/or environmental factors, may affect the function of the udder and thus milk productivity.

## 5 Conclusions

Our study provides an all-round insight into the udder functional morphology of Cyprus Damascus goats (ARI nucleus herd) in relation to milk performance, by thoroughly examining udder morphology and its association with relevant factors (i.e., age, litter size, rearing method, seasonal milk yield, and udder stage). Our findings can be viewed as an additional and novel, effective selection method to be examined in breeding programs, especially under the possibility of associating the indices with genetic data. Furthermore, this approach can be further developed to be applicable in younger Damascus goats and also in other breeds and species. Such development may provide an early, easy, and effective way to assist the genetic selection procedures in achieving higher milk production while mitigating the negative environmental and ethical concerns associated with livestock farming.

## Ethical statement

The recorded data did not involve any invasive measures. All of the animal handling and care during the recordings were applied in accordance with the Cyprus Law on the use of Animals in Scientific Experiments, 133(I)/2005.

## Declaration of generative AI and AI-assisted technologies in the writing process

No AI or AI-assisted technologies were used in the writing process of this article.

## Financial support statement

This study was funded as part of the nationally-funded AGRICYGEN project.

## Data and model availability statement

The data used in the study were not deposited in official repositories. The data can become available upon request from the corresponding author and authorization by the Agricultural Research Institute of Cyprus.

## CRediT authorship contribution statement

**P. Savvides:** Writing – review & editing, Writing – original draft, Visualization, Validation, Software, Methodology, Investigation, Formal analysis, Data curation, Conceptualization. **G. Hadjipavlou:** Writing – review & editing, Validation, Supervision, Resources, Project administration, Methodology, Funding acquisition, Conceptualization.

## Declaration of competing interest

The authors declare that they have no known competing financial interests or personal relationships that could have appeared to influence the work reported in this paper.

## Appendix Supplementary materials

Image, application 1
