# Supplementary material for: Exploring udder functional morphology in nucleus herd Cyprus Damascus goats and its effects on milk yield
Source: Vet Anim Sci. 2026 Jun 17;34:100743. doi: 10.1016/j.vas.2026.100743 (PMC13312077; doi:10.1016/j.vas.2026.100743)
Supplement: Supplementary file 1 [file mmc1.docx]

| **Characters** | **Age group A (n=19)** | **Age group B (n=56)** |
| --- | --- | --- |
| **WL** | 70.4 | 70.86 |
| **BL** | 80.32 | 82 |
| **HL** | 26.53 | 27.5 |
| **CL** | 14.45 | 15.53 |
| **TL** | 17.45 | 17.7 |
| **URD** | 15.93 | 20.14 |
| **UD** | 9.1 | 4.83 |
| **UC** | 35.32 | 36.37 |
| **RTL** | 3.89 | 3.8 |
| **LTL** | 4.03 | 4.07 |
| **RTC** | 6.33 | 6.35 |
| **LTC** | 6.59 | 6.59 |
| **DBT** | 13.7 | 15.56 |
| **UH** | 32.44 | 28.9 |
| **TH** | 29 | 24.76 |
| **UV** | 5175 | 6732.9 |
| **Milk yield total** | 169.56 | 189.03 |
| **Milk yield 60** | 80.74 | 94.35 |
|  |  |  |

Supplementary table. The averaged values of each quantitative morphological character for both age groups. Description of each character from and abbreviations: 1. height at the withers, HW (ICAR 2022), 2. body length, BL (Esquivelzeta et al., 2011), 3. head length, HL (Esquivelzeta et al., 2011), 4. chest width, CW (ICAR, 2022) and 5. tail length, TL (Aparicio, 1944). Description of each character from Fig. 1b and abbreviations: 1. udder depth, UD (ICAR, 2022), 2. udder rear depth, URD (Emediato et al., 2008), 3. udder circumference, UC (Emediato et al., 2008), 4. right and left teat length, RTL and LTL (Mavrogenis et al., 1989), 5. right and left teat circumference, RTC and LTC (Wang, 1989), 6. distance between teats, DBT (Wang, 1989), 7. udder height, UH (Wang, 1989), 8. teat height, TH, and 9. udder volume (UV) (Emediato et al., 2008). All morphological characters are in cm except UC (cm^3^) and Milk yield total and 60 (kg).
